# Categorising commercial sector practices to advance human and planetary health

**DOI:** 10.1093/eurpub/ckac131.543

**Published:** 2022-10-25

**Authors:** A Gilmore, A Fabbri, K Bondi, A Bertscher, J Lacy-Nichols

**Affiliations:** 1 Department for Health, University of Bath, Bath, UK; 2 School of Population and Global Health, University of Melbourne, Melbourne, Australia

## Abstract

**Background:**

There is growing interest in the commercial determinants of health, broadly understood as the ways in which commercial actors influence health & health equity. Such actors can impact on health & health inequity, positively or negatively, through two main routes: (1) producing & driving use of/access to products or services potentially damaging or beneficial to health. (2) through diverse commercial practices which range from marketing, through lobbying, to tax & labour practices. Practices are arguably of greater importance given the growing shift to private sector employment & because all industries, not just those whose products are harmful, engage in them. Yet their contribution to physical, mental & planetary ill health is generally under-recognised with a focus hitherto on unhealth commodities. We aim to develop a practical categorisation of commercial sector practices

**Methods:**

Narrative review & content analysis of existing categorisations (those attempting to cover all practices), & taxonomies (those focused on specific practices (e.g., marketing), related academic &grey literatures.

**Results:**

There is dualism between the business studies & public health literatures, the latter tending to dichotomise market & non-market strategies while the former sees this as an outdated approach. While some specific practices are well documented, others remain poorly understood & theorised. A simplified categorisation is proposed which attempts to identify key commercial sector practices that can impact on health, the ways in which they overlap & reinforce each other. Evidence-based taxonomies for specific practices are identified.

**Conclusions:**

Our categorisation provides a simple approach through which the commercial practices that can harm human & planetary health & drive health equity can be understood & addressed. It draws attention to the diverse ways through which corporations can harm health, identifies areas for action & further research.

**Key messages:**

• The ways in which commercial sector practices influence health and health equity are diverse yet poorly understood.

• We offer a simple categorisation of commercial practices which draws attention to the diverse ways through which they can harm health, helps identify solutions and areas for PH action.

